# Pain: Behavioural expression and response in an evolutionary framework

**DOI:** 10.1093/emph/eoad038

**Published:** 2023-11-08

**Authors:** Amanda C de C Williams

**Affiliations:** Research Department of Clinical, Educational & Health Psychology, University College London, Gower St, London WC1E 6BT, UK

**Keywords:** pain, response, evolutionary, facial expression, behaviour, empathy

## Abstract

An evolutionary perspective offers insights into the major public health problem of chronic (persistent) pain; behaviours associated with it perpetuate both pain and disability. Pain is motivating, and pain-related behaviours promote recovery by immediate active or passive defence; subsequent protection of wounds; suppression of competing responses; energy conservation; vigilance to threat; and learned avoidance of associated cues. When these persist beyond healing, as in chronic pain, they are disabling. In mammals, facial and bodily expression of pain is visible and identifiable by others, while social context, including conspecifics’ responses, modulate pain. Studies of responses to pain emphasize onlooker empathy, but people with chronic pain report feeling disbelieved and stigmatized. Observers frequently discount others’ pain, best understood in terms of cheater detection—alertness to free riders that underpins the capacity for prosocial behaviours. These dynamics occur both in everyday life and in clinical encounters, providing an account of the adaptiveness of pain-related behaviours.

Pain is studied, and treated, in the context of increasingly well-understood proximate mechanisms, but with little attention to ultimate—evolutionary—considerations, beyond acknowledgement of the effective defensive function of acute pain. Chronic pain is a huge public health problem and the cause of substantial disability and distress, but healthcare practitioners often dismiss it as a dysfunction of an otherwise essential system (e.g. Ref. [1]). Appreciating the functions of pain-related behaviours offers greater understanding, with therapeutic implications. Most studies of pain behaviour in a social context concern humans. Other animals communicate and respond to pain in similar ways, but their pain can only be inferred from behaviour. After describing pain mechanisms essential for understanding behaviour modulation, pain-related behaviours and conspecific responses are described, mainly in rodents and in humans, discussing both affiliative and non-affiliative responses. A mismatch model of pain [2–4] offers a promising framework for reconceptualizing chronic pain, and addressing treatment shortcomings.

## NATURE AND FUNCTION OF PAIN

Pain is often described as a sensation, with a signal reaching an analytic brain that appraises it and organizes and executes a response. This model, implicit in much lay and medical language, is inaccurate and misleading. In contrast, ‘The [gate control] model suggests that psychological factors such as past experience, attention, and emotion influence pain response and perception’; ‘central nervous system activities subserving attention, emotion and memories of prior experience... exert control over the sensory input’ [5]; and ‘psychological factors can influence nociceptive processing at the earliest stage of the central nervous system, namely the dorsal horn of the spinal cord’ [6]. Unmodulated pain would actually disable an organism under attack in a profoundly maladaptive way: pain can be amplified or inhibited top-down [7], according to need.

These understandings refer to proximate mechanisms and have contributed to the definition of pain in humans: ‘An unpleasant sensory and emotional experience associated with, or resembling that associated with, actual or potential tissue damage’ [8], asserting the centrality of subjective experience and the importance of emotion. Definitions of pain for non-human animals propose behavioural signals that may be interpreted as pain. However, in many experimental settings, pain is evoked rather than spontaneous and subject to multiple constraints on both modulatory factors and responses (discussed below). Additionally, behaviours associated with (what we assume to be) the experience of pain can also indicate fear or sickness: there are no biomarkers of pain by which to distinguish these.

Separate definitions for humans and animals, while based on robust scientific work, have encouraged theorizing unique to humans and their capacities, with very little comparative ethology or even anthropology of pain. However, many pain mechanisms are highly conserved across species [9, 10]. To understand pain-related behaviours in individuals with pain and in those observing them, we need an evolutionary perspective that addresses the functions of pain in varied environmental and social contexts, and the trade-offs against other exigencies of survival.

Walters [11] describes function in terms of: immediate behaviours of reflex withdrawal, identification of location and severity of pain, and memorization of context and cues; rapid responses of passive or active defence, minimizing further damage and suppressing competing responses; and longer-term behaviours of wound protection and care to promote healing, conservation of energy, vigilance to threat and avoidance of similar cues and contexts.

These are enabled by mechanisms of sensitization at multiple levels, from peripheral nociceptors to brain circuits that become more active and reactive, producing heightened responsiveness [12]. A study in squid [13] has elegantly demonstrated this. Both injured (part of one arm removed) and uninjured squid were subject to predation by fish that preferentially targeted injured squid, but squid that had not been anaesthetized during injury, and so had sensitized arms, were more vigilant and initiated defensive behaviours earlier than those injured under anaesthetic and without sensitization. There was a clear and direct survival advantage to those with sensitization. Subsequent studies demonstrated that early injury in squid produced lifelong sensitization [14]. Sensitization itself is amplified or inhibited according to context, threat or safety cues, including social signals.

Plasticity in the pain system, therefore, provides mechanisms by which the organism can adjust its behaviour to the level of threat. Pain can be considered a motivational system to maintain or restore bodily integrity, so the injured animal needs to employ extra vigilance to prevent further injury or delayed healing, both in terms of threat from the environment and from infection of or further damage to wounds. Inflammation and mobilization of the immune system involve defences that include motivational and behavioural changes [15]. The extra vigilance can persist: mice with lasting neuropathic pain from inflicted injury are much more likely than their pain-free peers to take a longer route to a reward, rather than their habitual shorter route when they detect fox odour in the latter [16].

Pain can persist, failing to recover or to respond to analgesics and other interventions effective for acute pain. Although conventionally described as maladaptive and dysfunctional, the prolonged hypervigilance conferred by persistent pain may, in fact, be adaptive [17] in an environment estimated to be dangerous. However, the associated demotivation, suppression of appetitive behaviours and immobility also carry risks, particularly when prolonged. Many animals cannot go long without foraging or hunting and are more at risk when separated from their social or family group or herd. There is thus a trade-off between the pain-and-recovery inactive state and the activities required for survival. This has given rise to the ‘mismatch’ hypothesis of chronic pain, whereby, when survival needs can be met by conspecific help, as in many human societies, lack of activity itself may serve to prolong pain—in humans and in their domesticated, companion and captive animals [2, 3]. When activity is required, for escape or hunting or foraging, descending pain modulation is switched on to allow movement [4]. In humans, attention to and anticipation of pain on movement affect both behaviour, tending towards avoidance of activity, and expectations, possibly promoting pain amplification [18]. Evidence for the mismatch hypotheses is hard to acquire [19], but the effect of exercise in promoting recovery from pain in rodents is strongly supportive: physical activity (voluntary or forced) appears to counter sensitization and decrease pain [20], with evidence to support the extension of this principle to humans [20, 21].

Pain and sensitization, and related behaviours, subside once the organism is safe and healing or healed, but the processes involved have received surprisingly little attention. The role of helping—of provision of food, water and safety—by conspecifics, or by humans for domesticated and captive animals—is central to the mismatch hypothesis. The COVID pandemic demonstrated that it was possible for humans, in some circumstances, to survive without leaving the home for months or even years, through donated and/or purchased help. Curiously, help is more often described in terms of empathy or other motivation, or of characteristics of the help-giver, than by the cues that elicit it. Since pain may have no associated visible signs of injury or pathology, the behaviours that indicate pain to others are of particular interest, and are further explored below.

## COMMUNICATION OF PAIN

None of the behaviours listed above [11], although often visible to onlookers, appears to have a primarily communicative function. Expression of pain solely for communication costs energy and so must have benefits to the individual and/or to onlookers that outweigh those costs for it to persist over evolutionary time. Benefits must also outweigh the risks that competitors (conspecific or other) or predators might identify and exploit signs of vulnerability. Yet helping wounded conspecifics (other than by parents) has rarely and only anecdotally been recorded in non-human animals. Benefits to individuals in pain may be subtle and difficult to observe, as in social analgesia in rodents (described below); the benefit to onlookers of information about proximal threats is more evident.

In humans, care in the context of pain was first formulated and studied experimentally as operant behaviour, reinforced by rewards in the environment, particularly from other people [22, 23] but, the less tightly controlled the experimental setting and task, the weaker the evidence for operant control of pain-related behaviours [24–26]. Below, it is described in relation to roles, including stranger, familiar, partner and clinician.

## PAIN-RELATED BEHAVIOUR IN RODENTS

Much of what is recorded as pain-related behaviour in mice is elicited for the purposes of research on pain and analgesia, and there is active debate about how much evoked pain and elicited behaviours can inform us about human clinical conditions [27, 28]. For instance, paw withdrawal from a noxious stimulus (often a series of hairs of graded stiffness) has been criticized as being merely reflex indicating hypersensitivity rather than pain [29]. Models of thermal and mechanical hypersensitivity, rather than of spontaneous pain, dominate the research literature [29] and, although technology offers ways to capture subtler behaviours [30], the issue of what is being assessed remains. Spontaneous behaviours such as burrowing can be affected by pain [31], and have better ecological validity than evoked responses, but some behaviours such as inactivity and lack of grooming (as in cancer pain studies) may indicate sickness or pain or both [29]. Cognitive (e.g. attention, memory) tasks offer parallels with human pain studies [32], and assessment of quality of life has been proposed.

Signals that communicate pain to conspecifics (and researchers) occur in various modalities: vocalizations, olfactory signals and motor behaviours, including facial expression. In humans, there is a distinct facial expression of pain [33]; similar expressions using many of the same facial muscles have been identified and described in mice [34] and rats [35], reliable to the extent that they can be used for testing analgesics [36, 37]. There are now scales for many other mammals [36] (described below).

The social context of pain-related behaviours is important [29], including the stress induced in male mice by male human experimenters [38], a concern still inadequately addressed in rodent pain research. Of particular interest are social influences on pain and related behaviours in mice, more investigated than any other non-human mammal. Pain shown behaviourally by one mouse sensitizes cagemates that can see the mouse in pain, while the mouse in pain shows more pain-related behaviour with a littermate present than with a strange mouse [39–41]. Olfactory cues between mice can also confer hyperalgesia [41]. If a mouse in pain is in a ‘jail’, a free female mouse observing the mouse in pain will choose to stay close to it, appearing to reduce its pain by doing so [40]. Male mice have hierarchies and their response to a familiar mouse in pain is affected by position in the hierarchy [42] and by stress, such that mild social threat appears to produce hyperalgesia and strong social threat produces hypoalgesia or analgesia [43].

## ONLOOKER RESPONSES

Empathy, or emotional contagion, is often used to interpret mouse behaviour in the presence of another mouse’s pain [39, 44]. Rats will also act to terminate the distress of another rat [45], and there are observational accounts of many social mammals doing this for another adult conspecific, including elephants [46]. Consolation after conflict has been observed in mammals, including chimpanzees [47], bonobos [48] and dogs [49], and in some birds [50], but may serve functions other than reducing distress. A recent study [51] showed zebrafish that saw a conspecific in distress after injury tended to stay nearby, reducing the other’s distress; as in mammals, the behaviour was mediated by oxytocin. These behaviours are consistent with emotional contagion, but the behaviour of ants that rescue and tend the wounds of fellow ants injured in raids on termite nests, a behaviour controlled by pheromones [52], has not been described [53]. Studies of acute and persistent pain in other insects [54, 55] and invertebrates [56] have not yet investigated social factors that influence others’ behaviour.

According to De Waal and Preston [57], a model of empathy that applies across humans and non-human animals (i.e. not driven by cognitive mechanisms) builds on a core of motor mimicry. It involves the sharing of affective states (termed emotional contagion) and, at its most complex, perspective-taking and possibly providing help. Parental responsiveness to the needs of offspring enhances their survival [57, 58], as well as activating the parent’s reward system [58]. Maternal (or parental) response to distress in their young is widespread among mammals, and arguably in some birds [59], so is within their behavioural repertoire for potential extension to other conspecifics. Such help does not require perspective-taking, and responsiveness to the needs of the dependent young has to be balanced against the energy costs to the parent, so inhibition of emotional contagion, or of related action, may be necessary [60]. However, emotional resonance between animals does not necessarily result in helping with a cost to the help-giver. Targeted helping (more than simple cooperation) between adult conspecifics appears to be relatively rare, or perhaps rarely observed and appreciated, and surprisingly little attention is given to the exploitation of observed neediness of a conspecific, for instance, by challenging for status [60].

Distinct from motor behaviours and many vocalizations and olfactory signals, facial expressions are visible only to nearby conspecifics. Since any behaviour has an energy cost, facial expression is unlikely to be a simple readout of internal state, but a communication to those close [61, 62], most likely to be familiars. Following their development in mice [34], descriptions or standardized scales now exist for cats [63], sheep [64], cattle [65], piglets [66], horses and donkeys [67, 68], mice and rats (above), and other mammals, not least those used in analgesic studies [69]. Facial expression and observation of other pain behaviours are underused in the care of farm animals [70]. Facial expression appears to have a communicative intent, as suggested by maternal responses [71], and studies of dogs’ facial expressions (although not of pain) also strongly suggest communicative intent towards humans [72]. Because the presence of humans can suppress as well as elicit facial expressions, automated detection of pain facial expression is developing in horses [68] and sheep [73].

## PAIN-RELATED BEHAVIOUR IN HUMANS

Historically, pain-related behaviours in humans, with more focus on gross motor behaviours, such as limping and guarding, than on facial expression, were characterized as contingent on reinforcements in the environment, particularly social reinforcement [22]. While responsiveness to reinforcement is demonstrable in controlled settings (e.g.Ref. [25]), the operant framework cannot explain many phenomena around pain communication in humans. Researchers later proposed a distinction between protective and communicative pain behaviours [74], but both were poorly defined and not inherently distinct, since any visible behaviour may communicate the agent’s state, intentionally or not.

The notion of social analgesia, as in mice, has so far had little traction. In human infants, skin contact is demonstrably analgesic [75], as is soothing touch [76], both investigated during painful medical procedures (such as blood sampling by heel puncture). In adults [77], soothing touch, particularly from a trusted familiar, reduces experimentally produced pain, but its relevance for acute or chronic clinical pain is unclear. As described above, pain has an essential emotional component: this is minimal in most experimental paradigms but, in clinical settings, anxiety about pain may be paramount and responsive to social cues, as is the depressed affect in chronic pain [10, 22].

The notion of social analgesia in humans is plausible since the presence of another can signal safety and possible help, activating descending inhibition of pain. Pain may be suppressed in front of strangers, including experimenters in research studies; suppression in medical settings is likely more variable, given that the clinician, while usually a stranger, occupies a familiar helping role. A review by Krahé *et al.* [78] of social modulation of pain, albeit in experimental settings, showed that modulation depended on the relationship of the other person to the individual in pain, and the apparent intentions and agency of social partners; these factors were interpreted as signals (of variable reliability) of the other person as safe or threatening, an explanation supported by an independent study [79]. The suppression of pain-related behaviour in front of strangers, and its release in the presence of familiars or presumed allies, is particularly interesting: it turns on its head the common assumption that when an individual in pain is observed to amplify pain-related behaviour in the clinician’s presence, it indicates wilful and possibly duplicitous exaggeration [80]. Despite the substantial overlap with social aspects of placebo analgesia [81, 82], social analgesia in adult humans in clinical settings remains to be systematically investigated.

In terms of health and impact on life, chronic (persistent) and episodic pains, such as low back pain, osteoarthritis and rheumatoid arthritis, inflammatory bowel disease, migraine and many others, are of far more clinical concern than acute pains that can often be managed pharmacologically. It is unclear to what extent pain-related behaviours habituate as pain becomes chronic. Pain-related behaviours are effortful (e.g. limping) or hard to fake (e.g. facial expression), and reliably recognized [33]. The facial expressions characteristic of acute pain tend to be seen only in acute exacerbations of chronic pain, with perhaps very subtle aspects of the expression identifiable only by observant familiars [33]. Other behaviours such as sparing and guarding the affected part, for example, limping to reduce weight-bearing, may be functional in preventing pain exacerbation, but can also attempt to hide pain even from close others [83]. Vocalizations, verbal and paraverbal may be communicative but can indicate effort, pain, both or neither. In chronic pain, vigilance and caution are underpinned by sensitization processes, as demonstrated for squid [13] and for mice [16]. Vigilance in humans with chronic pain, however, is often described in pathological terms as ‘hypervigilance’ [84].

Pain-related behaviours show substantial overlap with sickness behaviours [15], and with behaviour in depression [10, 85], including conservation of resources, withdrawal from social contact and loss of interest in previous sources of interest and pleasure. These motivational deficits are also evident in mice and other animals with chronic pain [86]. Identifying and attempting to understand common behaviours and their proximate and ultimate functions [84] could usefully draw on understanding of sickness behaviour [15] and the diverse evolutionary theories proposed for depression (e.g. [87–91]).

## RESPONSES OF OTHER PEOPLE TO PAIN-RELATED BEHAVIOURS

First, it is important to recognize that pain-related behaviours may be suppressed in the presence of social threat [92], strangers and in adverse environments. Clinical environments represent a mixture of threat and safety, depending on the previous experiences of individuals with pain, their expectations and the behaviours (and beliefs) of clinical personnel. Studies of others’ responses to expression of acute or chronic pain have mostly involved spouses or partners, parents of children or clinicians.

Within an operant model of pain-related behaviour [23], studies of sequential interactions between people with chronic pain and their spouses during household tasks [24] showed that spouses’ solicitous behaviours not only consistently followed nonverbal pain behaviours but also preceded them, while spousal aggression appeared to suppress pain behaviour. The association of greater disability with more frequent solicitous spouse behaviour [23] was routinely interpreted as being fostered by this, but more disabled people may elicit more frequent help from their spouses. More solicitous spouses tend to be less distressed and report higher marital satisfaction than non-solicitous ones [93]. Similar dynamics occur in studies of children with chronic pain and their relationships with parents: higher levels of parental worry and protective behaviour are associated with greater child disability [94]. Newton-John [95] observed that many solicitousness studies that provided support for operant models of pain defined solicitousness using researchers’ expectations of the reinforcement value of (often instrumental) helping responses, not observed consequences or self-report by recipients. These findings contrast with those in health, in general, where social support promotes health in humans [96, 97] and other social animals [98].

Instead of direct observation, most studies of clinicians’ judgements of people with pain use patient photos, videos or written accounts of behaviour. The commonest data elicited are estimates of pain intensity, with a very consistent tendency for clinicians to underestimate it [99]; this tendency is more marked with female patients, higher expressed pain, more years of clinician experience, no obvious cause of pain and any psychological disorder or psychiatric history [100, 101]. For their part, people with chronic pain consistently describe not feeling understood even by those close to them, being viewed with suspicion and being stigmatized [102, 103]. This makes sense in terms of human prosociality and a tendency to cooperation that is balanced by alertness to possible exploitation [104]. Invisible disabilities such as chronic pain are easily subject to such suspicion.

## HUMAN PAIN AND SOCIALITY

When considering human pain and sociality, pain-related behaviours, onlooker responses and the social factors that influence them in both humans and non-human animals all need to align with our understanding of pain mechanisms. While an implicit stimulus-response (noxious stimulus pain behaviour) model dominates much medical and psychological formulation of pain, a more accurate model is of active inference by a Bayesian brain of imminent sensory events, and processing in the context of actual sensory input, with difference (‘error’) minimized by updating of expectations or action to changed sensory input [82, 105]. Pain is thus the result of a prediction error [106], with particular salience where the individual is already sensitized to threat. An anxious individual who overestimates risk and misses opportunities to disconfirm predicted harm will maintain those prior expectations [107]. These inferences are implemented through ascending and descending modulatory pathways [108], and determine motivations and relative priorities [109].

Humans share with other social animals a sensitivity to social stresses that affects the inflammatory system [110], as well as the pain system. However, humans are unique in the extent to which they are socially engaged with others [104], conceptualized by some as central in brain development and function [111, 112]. Social distress may even have co-opted pain-processing pathways [10, 113]. Pain often, but not always, evokes empathic responses in onlookers, but it is important not to disregard inadvertent callousness, as in clinical underestimation of pain, and deliberate and extreme cruelty, as in torture and other abuse. Agent-based modelling can be used to simulate how visibility of pain and observer responses interact, starting with different population densities of showing/not showing pain and helping/ignoring/exploiting others in pain [114]. Somewhat surprisingly, even when expressing pain was extinguished, the capacity for altruism persisted, and frequency of helping was relatively unresponsive to changes in costs or benefits of helping. By contrast, increased interactions between agents in pain and potential helpers diminished benefits to both. Although this was inevitably a highly simplified exploration, it can help to suggest relevant dynamics. Pain disrupts fundamental social needs [115], and reconnection through behaviour that elicits empathic responses and instrumental help may contribute to apparent social analgesia. There is much we do not know about the influence of social and environmental factors on expressiveness in pain, and about responses to it, particularly beyond family and clinical settings. In non-human animals, where instrumental help is very rare, it is harder to model adaptive mechanisms of signalling pain, but we may only have started appreciating mechanisms of social analgesia.

## CONCLUSION

Understanding the functions of behaviours associated with pain, and what facilitates and inhibits them, generates previously unconsidered routes to reducing the problems of chronic pain. If the mismatch model is correct, early and regular activity should prevent acute pain becoming chronic by reducing the state of sensitization that characterizes acute pain. Like sickness behaviour [116], pain-related behaviours signal need which, in prosocial humans, motivates their help: those who help others may in turn be more reliably helped, consistent with kin and reciprocal altruism. Yet type and timing of help may need to promote, rather than delay, return to activity for the individual in pain. Questions about the evolutionary functions of pain-related behaviours, particularly long term, and about the variables that control them in the ancestral and modern environments, have potentially important implications for prevention of chronic pain.
